# Individual and Contextual Factors Associated With Maternal and Child Health Essential Health Services Indicators: A Multilevel Analysis of Universal Health Coverage in 58 Low- and Middle-Income Countries

**DOI:** 10.34172/ijhpm.2021.121

**Published:** 2021-09-01

**Authors:** Seun S. Anjorin, Abimbola A. Ayorinde, Oyinlola Oyebode, Olalekan A. Uthman

**Affiliations:** ^1^Warwick-Centre for Global Health, Division of Health Sciences, Warwick Medical School, University of Warwick, Warwick, UK.; ^2^Warwick-Centre for Applied Health Research and Delivery (WCAHRD), Division of Health Sciences, Warwick Medical School, University of Warwick, Warwick, UK.

**Keywords:** Universal Health Coverage, Essential Health Services, Low- and Middle-Income Countries, Multilevel Analysis

## Abstract

**Background:** Universal health coverage (UHC) is part of the global health agenda to tackle the lack of access to essential health services (EHS). This study developed and tested models to examine the individual, neighbourhood and country-level determinants associated with access to coverage of EHS under the UHC agenda in low- and middle-income countries (LMICs).

**Methods:** We used datasets from the Demographic and Health Surveys (DHSs) of 58 LMICs. Suboptimal and optimal access to EHS were computed using nine indicators. Descriptive and multilevel multinomial regression analyses were performed using R and STATA.

**Results:** The prevalence of suboptimal and optimal access to EHS varies across the countries, the former ranging from 5.55% to 100%, and the latter ranging from 0% to 90.36% both in Honduras and Colombia, respectively. In the fully adjusted model, children of mothers with lower educational attainment (relative risk ratio [RRR] 2.11, 95% credible interval [CrI] 1.92 to 2.32) and those from poor households (RRR 1.79, 95% CrI 1.61 to 2.00) were more likely to have suboptimal access to EHS. Also, those with health insurance (RRR 0.72, 95% CrI 0.59 to 0.85) and access to media (RRR 0.59, 95% CrI 0.51 to 0.67) were at lesser risk of having suboptimal EHS. Similar trends, although in the opposite direction, were observed in the analysis involving optimal access. The intra-neighbourhood and intra-country correlation coefficients were estimated using the intercept component variance; 57.50%% and 27.70% of variances in suboptimal access to EHS are attributable to the neighbourhood and country-level factors.

**Conclusion:** Neighbourhood-level poverty, illiteracy, and rurality modify access to EHS coverage in LMICs. Interventions aimed at achieving the 2030 UHC goals should consider integrating socioeconomic and living conditions of people.

## Background

**Key Messages**

** Implications for policy makers**

- Universal health coverage (UHC) remains a critical global health policy to advance public health in low- and middle-income countries (LMICs).
- Policy-makers and relevant stakeholders must begin to focus on long-term policies and interventions to address the unacceptably high prevalence of poor access to health services in LMICs.
- While demand-side healthcare rationing continues to address immediate healthcare challenges in LMICs, policies that will advance girl’s education, reduce poverty, and strengthen community support are recommended as a long-term policy focus.
- Finally, policy-makers in LMICS should prioritise the living condition of citizens as a strategy to tackle health problems.

** Implications for the public**

This is an exciting time in global public health as world leaders, policy-makers, and stakeholders, including foreign donor agencies, agreed on how best to tackle the health challenges in low-resourced countries. It is called the universal health coverage (UHC) agenda. Our research explored one of the most critical aspects of this agenda: access to essential health services (EHS). One of the profound findings is that, beyond individual-level factors, neighbourhood factors - meaning the condition in which people live - has more impact on their access to health services. This research also emphasised the importance of investment in girl’s education as a critical strategy for health and human development at individual, community, and country levels. Finally, the findings from this study enable robust discussions and highlight new research themes related to access to EHS in low- and middle-income countries (LMICs).

The concept of expanding health service coverage to everyone and providing financial protection has been in existence since the 20th century.^1,2^ The German social health insurance scheme, founded in 1978, and the British National Health Service, founded in 1948, were the founding models; more countries, especially in Europe, adapted different models to design their health systems years after.^3,4^ The concept gained more attention in 2005 when the World Health Organization (WHO) mobilised all its member states to commit to advancing the concept in their respective countries.^5^ It is currently referred to as universal health coverage (UHC).^1^ It is broadly defined as access to essential and quality health services needed (referred to as health service coverage) without being exposed to financial risk.^1,6^

 UHC has emerged as one of the most critical post-2015 global health priorities; it is termed the most potent strategy to deal with public health issues and forms part of Sustainable Development Goal (SDG) indicators.^7,8^ Therefore, this concept has enjoyed massive support from global development agencies such as The World Bank, WHO and the United Nations. More than 100 countries, irrespective of their developmental phase, are on track to achieving UHC.^6^ They have received funding and technical support from these agencies to design and implement interventions to advance progress towards UHC; the global coverage of health services is it the highest of all times in history.

 Despite the progress made, a recent monitoring report revealed that more than 50% of the world’s population still lacks access to essential health services (EHS), with the highest prevalence in Southern Asia and Africa. At the same time, about 100 million are pushed into extreme poverty because of out-of-pocket health expenditure.^9^ This highlights the enormous work and tenacity needed at all levels of government to achieve UHC’s 2030 goal of 80% health service coverage.

 Though the UHC agenda has enjoyed great acceptance and consensus since its ascension to the global health priorities, clarifications on some conceptual and implementation strategies are required. Evidence on how countries should pursue and achieve UHC goals and how to track country’s progress were some of the key ambiguities.^1,5^ While some countries are currently focused on financing schemes through the implementation of health insurance, some others have emphasised the expansion of access to basic or selected health services free or at a reduced cost. Nevertheless, it has been highlighted that for countries to achieve the UHC agenda; interventions must involve both financing mechanism and expansion of health services coverage.^3,10,11^

 The World Bank and WHO, in 2017, developed a monitoring framework that enables smooth and comparable tracking of UHC progress across countries. Since then, some studies have examined UHC progress, especially access to EHS, using this framework; nevertheless, these were all done at the country level.^7,12,13^ These will not allow cross-country comparison. Those who managed to do so are predisposed to an ecological fallacy; therefore, their findings are limited in informing policies and practices needed to advance the UHC goal. We believe there is a need to use individual-level data to examine the correlates of access to EHS under the UHC umbrella. This will better inform policy design and implementation of interventions aimed at driving UHC goals. One of the objectives of this study was to fill this research gap by conducting a multilevel modelling analysis to identify factors associated with access to the health service component of UHC. Besides, there has been increased attention to how contextual factors, ie, where people live, socioeconomic and sociocultural constructs, influence health determinants and outcomes.^14,15^ Therefore, this study also examined neighbourhood and country-level factors associated with access to EHS within UHC.

## Methods

###  Study Design and Data Collection

 This study is based on data from the Demographic and Health Survey (DHS); data on demographic, environmental, socioeconomic, nutritional and health factors are collected through a cross-sectional study design implemented by ICF International. The surveys are implemented every 5 years in 90 low- and middle-income countries (LMICs), and because the survey instruments are similar across countries, datasets are largely comparable. The dataset used for this study is the child recode component of the most recent DHS conducted in 58 LMICs between 2010 and 2018 as of January 2020 (see Table S1 of Supplementary file 1 for more details).

 The DHS uses a three-stage stratified cluster design to select participants for their surveys, with households serving as the sampling units. Data were collected by face-to-face interviews with women who met the eligibility criteria; participation rates are usually more than 95%. Women are generally asked to provide a detailed history of all their live births in chronological order. It includes whether the delivery was single or multiple, assigned sex of the child, date of birth, survival status, age of the child on the date of interview if alive and age at death of each live birth, if the child was not still alive. More details on data collection procedures have been published elsewhere.^16^

###  Outcome Variable

 The outcome variable, access to EHS, was computed based on nine prevention and treatment indicators adapted from the WHO and The World Bank’s monitoring framework on UHC.^17^ The following prevention indicators were used: family planning, four antenatal care (ANC) visits, Bacille Calmette-Guérin (BCG) immunisation, three doses of diphtheria-pertussis-tetanus third-dose (DPT3) immunisation, measles immunisation, and use of insecticide-treated nets. Also, three treatment indicators included were skilled birth attendance, oral rehydration therapy (ORT) for childhood diarrhoea and acute respiratory infection treatment for childhood pneumonia (see Table 1 for their definitions).

**Table 1:** Universal Health Coverage Health Service Prevention and Treatment Indicators

**Prevention Indicators**	
Family planning needs satisfied	The proportion of currently married women aged 15-49 who do not want any more children or want to wait two or more years before having another child are using contraception.
ANC4+	The proportion of women aged 15-49 years in the five years preceding the survey who received at least four visits from a skilled health provider during their last pregnancy.
BCG immunisation	The proportion of children aged 12-23 months who received one dose of BCG vaccine.
DPT3 immunisation	The proportion of children aged 12-23 months who received three doses of diphtheria, pertussis and tetanus.
Measles immunisation	The proportion of children aged 12-23 months currently vaccinated against measles.
Insecticide-treated net	The percentage distribution of children 12-23 months with insecticide-treated nets.
**Treatment Indicators**	
Care seeking for pneumonia	The proportion of children under five years of age with suspected pneumonia (cough and difficult breathing NOT due to a problem in the chest and a blocked nose) in the two weeks preceding the survey taken to an appropriate health facility or provider and received the antibiotic treatment.
ORT treatment	The proportion of children under five years of age with diarrhoea in the last two weeks receiving ORS fluids made from ORS packets or pre-packaged ORS fluids.
Skilled birth attendance	The proportion of live births assisted by a skilled health provider (doctor, nurse, and midwife) in the 5 years preceding the survey.

Abbreviations: ANC4+, at least four antenatal care visits; DPT3, diphtheria-pertussis-tetanus third-dose; BCG, Bacille Calmette-Guérin; ORT, oral rehydration therapy; ORS, oral re-hydration solution.

 We defined the outcome variable as a categorical variable. Suboptimal access to EHS defined as when a child (aged 12-23 months) and mother (aged 15-64 years) pair has received three or fewer of the nine indicators. Optimal access was defined as when the mother-child pair have access to 6 or more of the nine indicators; and average access (used a reference group), as those with 4-5 of the nine prevention and treatment indicators. These cut-off points were determined based on explorations of the dataset by computing the interquartile range for access to UHC; the lowest was three, and the highest was 6. Similar cut-off points were used in the most recent global monitoring report on UHC.^17^

###  Individual-Level Variables

 We included the following factors as control variables: study year (categorised into <2014 and ≥2014), maternal age of marriage and religion. Other individual-level variables included in the models are maternal education, maternal age, employment status, whether a mother has health insurance schemes or not, access to media (television, radio, and newspapers) if a female-headed household. Also, the DHS has no information on household income; therefore, wealth index is being used as a proxy indicator to measure the respondent’s socioeconomic status. It was constructed using principal components analysis based on the following household variables: number of rooms per house, ownership of a car, motorcycle, bicycle, fridge, television, and telephone, as well as any kind of heating device.^18^

###  Neighbourhood Variables

 We conceptualised neighbourhoods as respondents sharing a common primary sampling unit within the DHS dataset. The DHS usually uses the most recent census of each country to compute the sampling frame and used it to identify primary sample units. The sample size from each cluster is usually optimum with high precision and, the primary sample unit is the most consistent across all surveys. Therefore, it is most suitable to identify neighbourhood when considering cross-region comparison as we have in this study. Thus, the following neighbourhood factors were included in the study: neighbourhood rurality, unemployment rate, illiteracy prevalence, and poverty level. We classified each of these factors into low and high to allow for non-linear effects and provide more readily interpretable results in the policy arena.

 Also, we examined ethnic diversity as one of the neighbourhood factors in this study. The ethnicity of the children was computed using the ethnicity diversity index formula (see the equation below). It also captures both the number of different ethnic groups in an area and the relative representation of each group.


$$Ethnic\;diversity\;index=1-\sum_{i=1}^{n}{\left[\frac{x_{i}}{y}\right]}^{2}$$

Where: *x*_i_ = population of ethnic group *i* of the area, y = total population of the area, n = number of ethnic groups in the area.

 Scores can range from 0 to approximately 1; however, for clarity of interpretation, each diversity index is multiplied by 100. The higher the index score, the greater the diversity in an area. It is zero if an entire population in a neighbourhood belongs to one ethnic group; if an area’s entire population belongs to one ethnic group, then the area has zero diversity. An area’s diversity index increases to 100 when the population is evenly divided into ethnic groups.

###  Country-Level Variables

 Human development index (HDI) and domestic government health expenditure were included in our model as the country-level variables. The HDI is a summarised measure of three key elements of human development, namely: standard of living (measured by gross national income per capita of each country), health (measured by life expectancy) and access to education (measured by average year of school by an adult and expected years of schooling for children of school entering the age). Principal components analysis was applied to the country-level data from these three dimensions to compute the index. The HDI was collected from the United Nations Program Report; we, in turn, used two tertiles to split data into three groups and categorised them into high, average, and low for easy interpretation. The domestic general government health expenditure reflects the government health spending from domestic sources in relation to each country’s economy-measured by gross domestic product. The data was extracted from the World Bank Development Indicators.

###  Statistical Analysis

 We performed descriptive, univariable analysis of individual-level variables and multinomial multilevel logistic regression to examine the individual, neighbourhood and country-level factors associated with suboptimal and optimal access to UHC relative to average access. The descriptive analysis result was presented in percentages. Data representation was adjusted for sample weight, stratification, and clustering.

 A three-level multinomial regression model for outcome reporting access to EHS was specified for child-mother pairs (level 1) within a neighbourhood (level 2) living in a country (level 3). We constructed five models; the first is an empty model, ie, without any explanatory variable; this was done to identify the level of variance between neighbourhood and country levels. The second model contains the individual-level variables while adjusting for the control variables; the third has the neighbourhood-level variable. The fourth model contains the country-level variables, and the fifth model has all the variables at all levels while simultaneously adjusting for each other. Finally, we performed sub-analyses by conducting separate multilevel analyses with a dataset containing only low-income countries (LICs) and middle-income countries (MICs). The result from these analyses is in Supplementary file 1.

 Relative risk ratio (RRR) at 95% credible interval (CrI) was used to report the associations between the variables. However, the intra-class coefficient and median odd ratio (MOR) were used to access variance; more details on these parameters are published elsewhere.^14,19^ Descriptive analyses were performed using R v3.6, and multilevel analyses were performed with the MLwin package in Stata 16^20^ using the Bayesian Markov Chain Monte Carlo procedure.

## Results

###  Descriptive

 This study involved analyses of data from 157 523 mother-child pairs (Level 1) living in 53 673 neighbourhoods (Level 2) from 58 LMICs (Level 3). The number of mother-child pairs ranged from 282 in South Africa to 50 857 in India. Table S1 shows a brief description of DHS data used per country, survey year and other characteristics of the surveys. We observed a wide variation in mother-child pairs access to EHS. The lowest and highest percentage of suboptimal access to EHS were observed in Honduras (5.5%) and Colombia (100%), respectively. Similarly, the lowest and highest access to optimal health services were observed in Colombia (0%) and Honduras (90.36%), respectively. Figure provided a graphical representation of variation in suboptimal access to EHS across the 58 LMICs. In the pooled sample, as presented in Table 2, about half of the respondents were less than 18 years old when they married, 50% were in the 26-34 years age category at the time of the survey, about 30% had no formal education and belonged to the poorest quintile. Also, only 11% have access to television, radio and magazine, 12% were from households headed by females, 33.6% were not working, and only 11% had health insurance.

**Figure:**
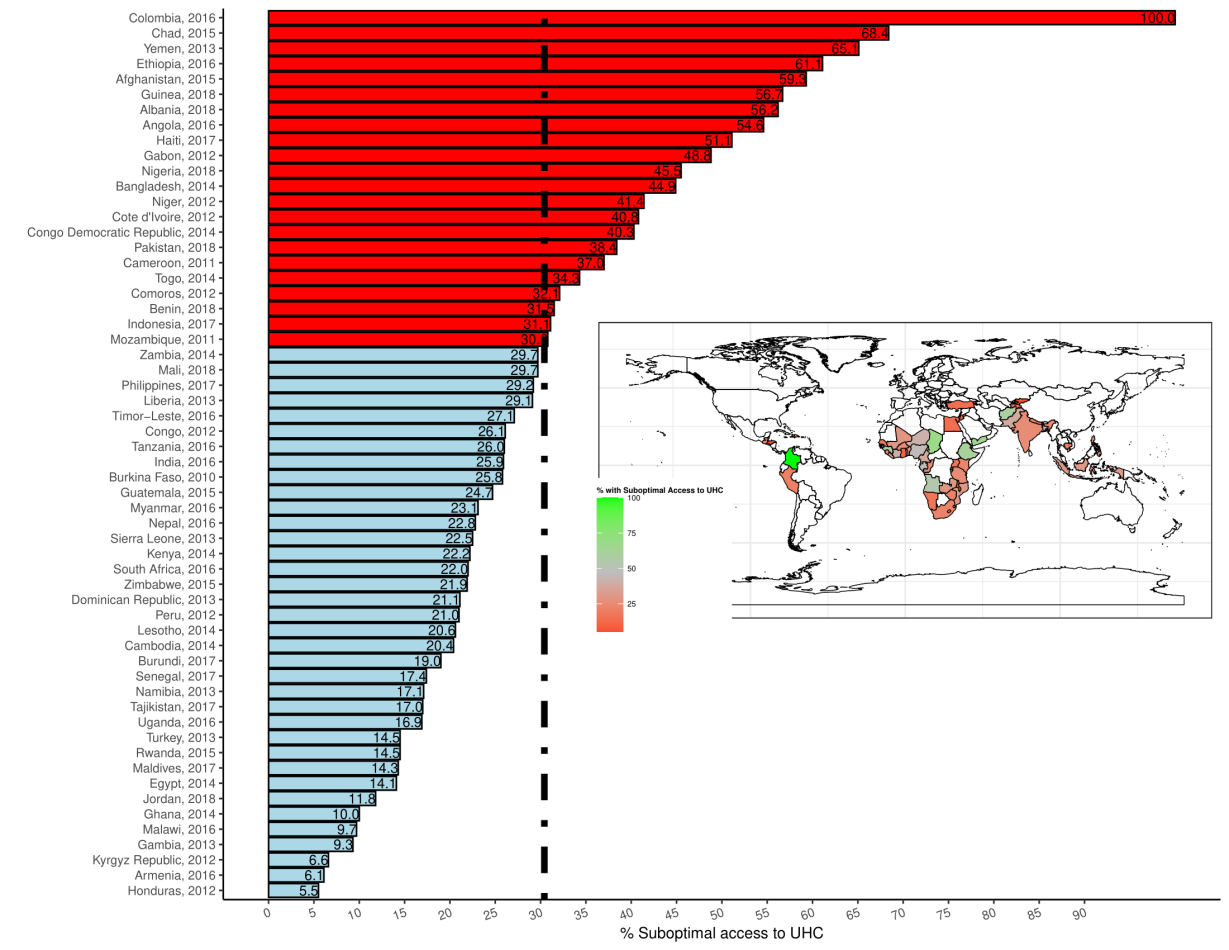
Graph and Map Showing Percentages of Suboptimal Access to EHS in LMICs. Abbreviations: UHC, universal health coverage; LMICs, low- and middle-income countries; EHS, essential health services.

**Table 2:** Descriptive Statistics With the Pooled Sample of Characteristics of DHS Data in LMICs

**Variables**	**Overall**	**Low Access**	**Medium Access**	**High Access**	***P*** ** Value **
Total	157 523	49 092	81 437	26 994	
Age of marriage (≥18)	73 465 (46.6)	18 540 (37.8)	40 849 (50.2)	14 076 (52.1)	<.001
Religions					<.001
Christians	48 663 (38.1)	13 414 (36.3)	24 819 (37.2)	10 430 (43.4)	
Muslim	33 945 (26.6)	12 171 (33.0)	16 093 (24.1)	5681 (23.6)	
Others	42 764 (33.5)	10 441 (28.3)	24 679 (37.0)	7644 (31.8)	
No religion	2375 (1.9)	895 (2.4)	1193 (1.8)	287 (1.2)	
Mother's age (y)					
14-25	54 262 (34.4)	16 757 (34.1)	27 665 (34.0)	9840 (36.5)	
26-34	78 699 (50.0)	23 443 (47.8)	41 715 (51.2)	13 541 (50.2)	
34-49	24 562 (15.6)	8892 (18.1)	12 057 (14.8)	3613 (13.4)	
Maternal education					<.001
No Education	51 213 (33.2)	23 009 (48.9)	21 991 (27.3)	6213 (23.1)	
Primary Education	37 933 (24.6)	11 256 (23.9)	19 874 (24.7)	6803 (25.3)	
Secondary Education	65 218 (42.2)	12 773 (27.2)	38 583 (48.0)	13 862 (51.6)	
Wealth					<.001
Low	52 197 (33.1)	21 911 (44.6)	23 903 (29.4)	6383 (23.6)	
Average	52 665 (33.4)	16 367 (33.3)	27 186 (33.4)	9112 (33.8)	
High	52 661 (33.4)	10 814 (22.0)	30 348 (37.3)	11 499 (42.6)	
Media access, mean (SD)	1.20 (0.98)	0.94 (0.97)	1.30 (0.97)	1.38 (0.95)	<.001
Maternal Female-head	20 215 (12.8)	5785 (11.8)	10 471 (12.9)	3959 (14.7)	<.001
Unemployed mothers	53 002 (33.6)	17 801 (36.3)	27 502 (33.8)	7699 (28.5)	<.001
Maternal had health insurance	17 876 (11.3)	3287 (6.7)	11 177 (13.7)	3412 (12.6)	<.001
Community-level poverty	78 013 (49.5)	29 621 (60.3)	37 035 (45.5)	11 357 (42.1)	<.001
Community-level illiteracy	69 835 (44.3)	27 103 (55.2)	32 840 (40.3)	9892 (36.6)	<.001
Community-level unemployment	64 544 (41.0)	20 122 (41.0)	33 383 (41.0)	11 039 (40.9)	.958
Diversity at community level	45 377 (28.8)	13 690 (27.9)	22 414 (27.5)	9273 (34.4)	<.001
Community-level rurality	4159 (2.6)	1959 (4.0)	1955 (2.4)	245 (0.9)	<.001
HDI					<.001
Low	54 806 (34.8)	20 237 (41.2)	24 753 (30.4)	9816 (36.4)	
Average	80 309 (51.0)	22 515 (45.9)	42 528 (52.2)	15 266 (56.6)	
High	22 408 (14.2)	6340 (12.9)	14 156 (17.4)	1912 (7.1)	
Domestic government health expenditure					<.001
Low	82 832 (52.6)	26 755 (54.5)	41 151 (50.5)	14 926 (55.3)	
Average	23 218 (14.7)	9374 (19.1)	9745 (12.0)	4099 (15.2)	
High	51 473 (32.7)	12 963 (26.4)	30 541 (37.5)	7969 (29.5)	

Abbreviations: HDI, Human development index; LMICs, low- and middle-income countries; DHS, Demographic and Health Survey. *Note*: Data are No. (%) unless otherwise specified. Percentages might not add up to 100% due to rounding.

###  Measure of Association

Table 3 showed the result from the different models implemented in our multilevel analysis for suboptimal and optimal access to UHC. In the final adjusted model (Model 5), we simultaneously adjusted for the effect of control variables, individual, neighbourhood, and country-level variables. The following were found to be significantly associated (*P*< .05) with the relative risk of sub-optimal and optimal when compared to average access to UHC; survey year, maternal education attainment and current age, wealth index, health insurance status, access to media, age of marriage, religion, neighbourhood poverty, illiteracy, and rurality.

**Table 3:** Multinominal Multilevel Analysis of Suboptimal and Optimal Access Compared to Average Access

	**Model 1**	**Model 2**	**Model 3**	**Model 4**	**Model 5**
		**RRR (95% CrI)**	**RRR (95% CrI)**	**RRR (95% CrI)**	**RRR (95% CrI)**
**Fixed Effect Model**					
**Suboptimal vs Average Access to UHC**					
Year		1.21 [0.97, 1.50]	1.53 [1.11, 2.10]^b^	1.25 [1.02, 1.54]^a^	1.54 [1.08, 2.20]^a^
Maternal age of marriage (<18 as ref)		1.18 [1.15, 1.22]^c^	1.29 [1.25, 1.33]^c^	1.45 [1.40, 1.49]^c^	1.17 [1.14, 1.21]^c^
Religion (Christianity as ref)					
Muslim		1.18 [1.11, 1.26]^c^	1.20 [1.13, 1.28]^c^	1.45 [1.37, 1.54]^c^	1.12 [1.05, 1.19]^c^
Other religion		0.73 [0.68, 0.79]^c^	0.72 [0.67, 0.76]^c^	0.85 [0.79, 0.91]^c^	0.70 [0.65, 0.75]^c^
No religion		1.18 [1.06, 1.32]^b^	1.28 [1.14, 1.43]^c^	1.46 [1.30, 1.64]^c^	1.17 [1.04, 1.31]^b^
Mother's age (14-25 years as ref)					
26-34		0.94 [0.91, 0.98]^b^			0.95 [0.92, 0.99]^a^
34-49		1.12 [1.07, 1.18]^c^			1.14 [1.08, 1.20]^c^
Maternal education (Secondary education as ref)					
No education		1.96 [1.88, 2.05]^c^			1.78 [1.69, 1.86]^c^
Primary education		1.43 [1.37, 1.49]^c^			1.39 [1.33, 1.45]^c^
Maternal wealth-index (High as ref)					
Low		2.05 [1.95, 2.14]^c^			1.74 [1.65, 1.84]^c^
Average		1.46 [1.40, 1.53]^c^			1.35 [1.29, 1.41]^c^
Media access		0.83 [0.81, 0.85]^c^			0.85 [0.83, 0.87]^c^
Female household-head		0.98 [0.93, 1.02]			0.98 [0.93, 1.02]
Unemployed mothers		0.99 [0.95, 1.03]			1.00 [0.95, 1.05]
Maternal has health insurance		0.73 [0.69, 0.78]^c^			0.74 [0.69, 0.78]^c^
Community-level factors					
Community poverty-level			1.73 [1.65, 1.80]^c^		1.15 [1.10, 1.21]^c^
Community illiteracy level			1.84 [1.76, 1.93]^c^		1.34 [1.28, 1.40]^c^
Community unemployment level			1.01 [0.97, 1.05]		1.02 [0.98, 1.06]
Diversity at community level			0.92 [0.89, 0.96]^c^		0.96 [0.92, 1.01]
Community rurality level			1.78 [1.48, 2.14]^c^		1.51 [1.25, 1.82]^c^
HDI (Low as ref)					
Average				1.61 [1.11, 2.33]^a^	0.77 [0.62, 0.96]^a^
High				1.89 [0.84, 4.27]	1.83 [1.04, 3.20]^a^
Domestic government health expenditure					
Average				0.47 [0.37, 0.59]^c^	0.94 [0.62, 1.43]
High				0.34 [0.26, 0.44]^c^	0.48 [0.35, 0.66]^c^
**Optimal vs Average Access to UHC**					
Year		0.50 [0.30, 0.82]^b^	0.71 [0.50, 1.01]	0.99 [0.83, 1.17]	0.52 [0.31, 0.88]^a^
Maternal age of marriage (<18 as ref)		0.90 [0.87, 0.94]^c^	0.89 [0.86, 0.92]^c^	0.87 [0.84, 0.90]^c^	0.90 [0.87, 0.94]^c^
Religion (Christianity as ref)					
Muslim		0.83 [0.78, 0.89]^c^	0.80 [0.75, 0.85]^c^	0.74 [0.69, 0.79]^c^	0.84 [0.79, 0.89]^c^
Other religion		0.73 [0.68, 0.78]^c^	0.73 [0.69, 0.79]^c^	0.69 [0.64, 0.75]^c^	0.73 [0.68, 0.78]^c^
No religion		0.82 [0.71, 0.96]^a^	0.78 [0.68, 0.90]^c^	0.76 [0.66, 0.88]^c^	0.83 [0.72, 0.96]^a^
Mother's age (14-25 years as ref)					
26-34		0.88 [0.85, 0.92]^c^			0.88 [0.85, 0.92]^c^
34-49		0.80 [0.76, 0.85]^c^			0.80 [0.75, 0.85]^c^
Maternal education (Secondary education as ref)					
No education		0.69 [0.65, 0.73]^c^			0.71 [0.68, 0.75]^c^
Primary education		0.84 [0.80, 0.88]^c^			0.84 [0.80, 0.88]^c^
Maternal wealth-index (High as ref)					
Low		0.87 [0.83, 0.91]^c^			0.83 [0.78, 0.88]^c^
Average		1.00 [0.96, 1.04]			0.99 [0.95, 1.04]
Media access		1.10 [1.08, 1.13]^c^			1.10 [1.08, 1.13]^c^
Female household-head		1.07 [1.03, 1.13]^b^			1.08 [1.03, 1.13]^b^
Unemployed mothers		0.97 [0.93, 1.01]			0.97 [0.93, 1.01]
Maternal has health insurance		1.31 [1.24, 1.39]^c^			1.30 [1.23, 1.37]^c^
Community-level factors					
Community poverty-level			0.96 [0.92, 1.00]		1.14 [1.09, 1.19]^c^
Community illiteracy level			0.74 [0.71, 0.77]^c^		0.87 [0.84, 0.91]^c^
Community unemployment level			1.00 [0.96, 1.04]		1.00 [0.96, 1.05]
Diversity at community level			1.20 [1.15, 1.25]^c^		1.17 [1.13, 1.22]^c^
Community rurality level			0.76 [0.58, 0.99]^a^		0.83 [0.64, 1.08]
Country-level factors					
HDI (Low as ref)					
Average				0.50 [0.41, 0.61]^c^	0.84 [0.71, 1.01]
High				0.16 [0.06, 0.47]^c^	0.27 [0.14, 0.51]^c^
Domestic government health expenditure					
Average				1.53 [0.94, 2.49]	1.09 [0.66, 1.80]
High				1.01 [0.78, 1.31]	0.71 [0.41, 1.22]
**Random Effect Model**					
**Suboptimal vs Average Access**					
Country-level variance	2.30 [1.57, 3.37]^c^	2.02 [1.47, 2.77]^c^	2.07 [1.46, 2.94]^c^	2.18 [1.48, 3.22]^c^	1.83 [1.40, 2.41]^c^
VPC (%, 95% CrI)	25.64 [19.61, 32.80]	26.31 [21.04, 32.41]	26.42 [20.62, 33.23]	25.43 [19.21, 32.90]	24.51 [20.22, 29.63]
MOR (95% CrI)	4.25 [31.01, 5.76]	3.88 [3.18, 4.89]	3.94 [3.17, 5.13]	4.09 [3.19, 5.54]	3.63 [3.09, 4.40]
Community-level variance	3.38 [3.16, 3.62]^c^	2.37 [2.25, 2.50]^c^	2.47 [2.33, 2.61]^c^	3.10 [2.92, 3.28]^c^	2.34 [2.23, 2.46]^c^
VPC (%, 95% CrI)	63.34 [59.03, 68.01]	57.15 [53.01, 61.53]	58.06 [53.53, 62.81]	61.62 [57.20, 66.42]	55.94 [52.43, 59.73]
MOR (95% CrI)	5.78 [5.45, 6.14]	4.34 [4.18, 4.52]	4.48 [4.29, 4.67]	5.36 [5.10, 5.63]	4.30 [4.16, 4.46]
**Optimal vs Average Access**					
Country-level variance	2.63 [1.65, 4.19]^c^	3.22 [1.78, 5.82]^c^	2.56 [1.61, 4.05]^c^	2.15 [1.47, 3.13]^c^	2.29 [1.42, 3.68]^c^
VPC (%, 95% CrI)	34.03 [24.81, 44.63]	39.34 [26.61, 53.53]	33.92 [24.84, 44.32]	29.82 [22.74, 37.72]	31.60 [22.54, 42.12]
MOR (95% CrI)	4.70 [3.41, 7.05]	5.54 [3.57, 9.99]	4.60 [3.35, 6.82]	4.05 [3.18, 5.41]	4.24 [3.12, 6.23]
Community-level variance	1.81 [1.72, 1.91]^c^	1.69 [1.62, 1.76]^c^	1.69 [1.60, 1.79]^c^	1.78 [1.70, 1.87]^c^	1.67 [1.59, 1.76]^c^
VPC (%, 95% CrI)	57.41 [50.63, 64.02]	59.93 [50.83, 69.75]	56.30 [49.42, 63.91]	54.42 [49.12, 60.30]	54.62 [47.82, 62.33]
MOR (95% CrI)	3.61 [3.49, 3.74]	3.46 [3.37, 3.54]	3.46 [3.34, 3.58]	3.57 [3.47, 3.69]	3.43 [3.33, 3.54]
*N*	127743	127743	127743	127743	127743

Abbreviations: RRR, relative risk ratio; CrI, credible interval; HDI, Human development index, UHC, universal health coverage; MOR, median odd ratio; VPC, variance partition coefficient. **Control variables**: Study year, mater age of marriage and religion; **Model 1**: empty; **Model 2**; maternal education, employment status, whether a mother has health insurance schemes or not, access to media, if a female headed a household, and mother’s decision making power; **Model 3:** neighbourhood rurality, unemployment rate, the prevalence of illiteracy and poverty level; **Model 4: **HDI and Domestic government health expenditure as percentage of GDP; **Model 5**: Fully adjusted model containing significant variables from each model. ^a^ *P* < .05, ^b^*P* < .01, ^c^*P* < .001.

 Explicitly, children of mothers with no education and only primary school education had 78%, and 39% increased relative risk for suboptimal access to UHC (RRR 1.78, 95% CrI 1.69-1.86) when compared to those with secondary education or higher. Children from the most impoverished household also had a 74% increased relative risk for suboptimal access to UHC (RRR 1.74, 95% CrI 1.65 to 1.84). On the other side, increased access to media and mother’s usage of health insurance reduces the relative risk for suboptimal access to UHC health services by 17% (RRR 0.83, 95% CrI 0.81 to 0.85) and 26% (RRR 0.74, 95% CrI 0.69 to 0.79) respectively. Compared with children of mothers practising Christianity, those who are Muslims (RRR 1.12, 95% CrI 1.05-1.20) and have no religion (RRR 1.17, 95% CrI 1.04-1.31) had 12% and 17% increased relative risk for suboptimal access to UHC. We also observed a significant risk of suboptimal access across maternal age bins. At the same time, the older women (34-49 years) had a 14% increased risk of suboptimal success; women between 26-34 years showed 5% reduced risk compared with those mothers in 14-24 years. Mother-child pairs living in neighbourhoods with a higher prevalence of illiteracy, poverty and rurality have 34% (RRR 1.33, 95% CrI 1.28-1.40), 15% (RRR 1.15, 95% CrI 1.10-1.21), and 51% (RRR 1.51, 95% CrI 1.25-1.82) increased relative risk of suboptimal access relative to average access to UHC health service. Finally, in communities with higher diversity, mother-child pairs have a 4% reduced risk of suboptimal access relative to average access; however, community-level unemployment was not significant.

 When optimal vs average access to UHC were compared, we observed a direct opposite of what we observed in the sub-optimal vs average access findings above. Mother-child pairs with no formal education and from poorest households have 29%, and 17% reduced access to optimal health services. Also, access to media, health insurance, and female household-head increases assess to optimal UHC by 10%, 8% and 30%, respectively. Children of mothers practising Christianity and other religions are less likely to have access to optimal UHC. Children of mothers living in neighbourhoods with a higher prevalence of illiteracy and diversity have 13% reduced (RRR 0.88, 95% CrI 0.84-0.91) and 17% (RRR 0.84, 95% CrI 0.64-1.11) increased relative risk for optimal UHC health services. Interestingly, the risk of optimal access relative to average access increases by 14% in communities with a high poverty rate. Mother-child pairs in neighbourhoods with a higher prevalence of poverty had 1.02 times relative risk of suboptimal access. For country-level factors, we also found that living in countries with a high HDI index only increases the relative risk or chance for optimal access to UHC health services by 0.27 times while it increases relative risk for suboptimal access by 1.83 times. Finally, high domestic government health expenditure reduces the risk of suboptimal access by 62% (RRR 0.48, 95% CrI 0.35-0.66), while its effect was not significant when optimal access and average access were compared.

###  Measure of Variation

 The variation in the odd of suboptimal access to UHC health service across countries and neighbourhoods is shown in Table 3. We observed significant variation in the relative risk for suboptimal (σ^2^ = 2.30, 95% CrI 1.57-3.37) and optimal (σ^2^ = 2.63, 95% CrI 1.65-4.19) access to UHC across the 59 countries, and across the 53,673 neighbourhoods (σ^2^ = 3.38, 95% CrI 3.16-3.62) and (σ^2^ = 1.81, 95% CrI 1.72-1.91) respectively. Based on the intra-neighbourhood and intra-country correlation coefficient estimated using the intercept component variance, 55.9% and 24.5% of the variance in suboptimal access to UHC and 54.6% and 31.6% of the variance in optimal access UHC are attributable to the neighbourhood and country-level factors.

 The MOR analysis further supports the modulating impact of societal and neighbourhood context in access to UHC. Our fully adjusted model suggests that if a mother-child pair moves to a neighbourhood and country with a higher certainty of suboptimal access to health service coverage, the media odd of experiencing suboptimal access to health service coverage will increase by 4.30% and 3.63%, respectively. In the same vein, the median odd of experiencing optimal access to UHC increases by 3.43% and 4.24% when a mother-child pair moved to a neighbourhood and country with a higher certainty of optimal access to UHC.

###  Sub-analysis of Low-Income and Middle-Income Countries

 The findings in the sub-analysis with LICs and MICs separately had the same pattern as in the pooled sample involving the 58 LMICs with a few differences. The increased risk of suboptimal access among mothers aged 34-49 in the overall pooled sample (RRR 1.14, 95% CrI 1.08-1.20) persisted only in MICs (RRR 1.14 95% CrI 1.09-1.20) (see Supplementary file 1). The increased risk of suboptimal access among Muslims in the pooled estimate was no longer significant in MICs (RRR 1.03 95% CrI 0.97-1.10). The impact of domestic government health expenditure was not substantial in MICs. Children of mothers with no education had an increased risk of suboptimal access by 53% in LICs (RRR 1.53 95% CrI 1.39-1.68), as against 78% observed in the overall pooled estimate. Children of unemployed mothers in LICs had an increased risk of suboptimal access (RRR 1.07 95% CrI 1.00-1.14), while community rurality was no longer significant.

## Discussion

 The overall objective of this study was to use hierarchical-level data to explore the distribution of access to EHS components of UHC. More importantly, we investigated individual-level, neighbourhood-level and country-level factors associated with access to EHS within UHC in developing countries. To the best of our knowledge, this is the first study that used individual data to explore the determinants of UHC, as all previous studies have focused on country-level data alone.

 The results from our analyses showed that there are some levels of variation in both suboptimal and optimal access to UHC across the 58 LMICs included. More than 50% of mother-child pairs have access to only three or fewer health service indicators (ie, suboptimal access to UHC) in Chad (68.38%), Yemen (65%), Ethiopia (61%), Albania (56.24%). On the other hand, more than 80% of mother child-pairs had at least access to 5 of the 9-health service indicators (ie, optimal access to UHC) in Armenia (93.88%), Honduras (90%), Kyrgyz Republic (89.10%), and Gambia (88.59%). These descriptive findings suggest that mother-child pairs in former countries have less access to health service coverage. These countries are largely behind in achieving UHC goals than their counterparts in later countries. Globally, we are now into the fifth year of the global SDGs agenda. Achieving 80% health service coverage by 2030 is one of the critical SDG targets under health and wellbeing domain. However, with recent estimates showing that about half of the world population do not have access to basic healthcare, it reveals there is much work to do. Previous studies have also supported some of our findings; although the computing index method is different, we used a similar or subset of indicators. Countries such as Ethiopia and Chad were found to have lower UHC indexes. Honduras and Armenia were reported to have one of the highest UHC indexes for EHS coverage.^17,22^ Individual country studies have also corroborated a similar trend in the prevalence of EHS.^12,13^

 Our multilevel logistic regression analysis investigated the strength of associations between access to health service coverage of the UHC component and various determinants. After adjusting for the country, neighbourhood, and individual-level factors simultaneously, we observed that maternal education, household wealth status, access to media, health insurance, and deprived socioeconomic neighbourhood and country’s HDI were associated with the relative risk of access UHC health services. Living in a household headed by a female and living in communities with high ethnic diversity were explicitly associated with optimal access to UHC health services. We suggest mothers’ educational attainment might be one of the strongest determinants of access to UHC as it has the strongest association with both suboptimal and optimal access to UHC. Mothers with no education are 78% more predisposed to suboptimal access to UHC (ie, receiving three or fewer of the nine EHS), and 29% less likely to have optimal access to UHC (receiving five or more of the nine EHS). Other than this, previous studies have shown that maternal educational attainment has a direct and strong positive association with other determinants such as maternal decision-making power, access to media and age at marriage.^23-25^ Health-seeking behaviour is strongly correlated with educational attainment, as mothers with better education can access and process health-related information that might help them raise their children.^26^

 In many LMICs, health-seeking behaviour and utilisation are usually a huge problem even when health services are available and accessible; this is largely because several other sociocultural and socioeconomic factors influence and shape access and usage.^27-29^ In our study, mothers from neighbourhoods with a higher prevalence of poverty, illiteracy, and rurality had at least 15% increased relative risk for suboptimal UHC. Similarly, optimal access to UHC health services was associated with neighbourhood level of illiteracy and ethnic diversity. Hypothetically, individuals from the same neighbourhood usually experience similar health outcomes. This is underpinned by the fact that they are usually susceptible to the same contextual exposures ranging from culture, economy, politics, and climate.^14^ Therefore, our study strongly suggests that some neighbourhood-level factors modify access to EHS coverage in UHC, and this corroborates with similar studies conducted in LMICs.^15,26,30^

 We also found that mother-child pairs living in LMICs with high HDI have suboptimal access increased by only 0.27 times and increased optimal access to UHC by 1.83 times. This finding is unanticipated; countries with high HDI should reflect human development advancement, better living conditions, and better access to EHS. Few studies have reported similar non-linear relationships between HDI and health outcomes, especially at the individual level.^31,32^ Country-level data such as HDI are usually susceptible to ecological fallacy, and as such, interpretation at the country level do not apply to individuals who made up the countries. Besides, HDI does not account for the distribution of inequalities in each dimension across a population^33^; with most LMICs well recognised for their wide health inequalities gaps, we suggest this might be another source for our finding of the association between HDI and access to EHS as observed in our study.

 The results from the VPC also support our findings from the fixed-effect models at the country-level; a more significant percentage of variance in both suboptimal and optimal access is attributable to neighbourhood-level factors compared to the country-level factors. Besides, from the MOR computed in our final model, if a mother-child pair move to a neighbourhood and country with a higher certainty of suboptimal access to health service coverage, the median odd of experiencing suboptimal access to health service coverage will increase by 4.30% and 3.63% respectively. On the other hand, if they moved to neighbourhood and country with a higher certainty of optimal access to health service coverage, the median odd of experiencing optimal access will increase by 3.43% and 4.24%, respectively. These findings probably suggest that deprived neighbourhoods had more impact on suboptimal access to EHS than a wealthy neighbourhood.

 The results from the sub-analysis possibly reflect how global health issues such as poor access to EHS in LMICs have similar attributes with only slight differences between LICs and MICs. While lack of education remains the most potent determinant for access to EHS in both LICs and MICs, the impact in MICs is higher than in LICs. It is also interesting to note that domestic government spending on health seems relevant only in LICs. These differences might be linked to other systemic and structural issues we could not capture but mentioned in the WHO/World Bank framework. Finally, this study also supports previous studies highlighting the importance of increasing government expenditure on health to combat poor or inadequate access to health services and advancing UHC in developing countries.

## Conclusion

 The findings from our analyses revealed that the lack of education is the strongest determinant of access to EHS at the individual level. This study also provides evidence that contextual factors such as neighbourhood’s poverty, illiteracy, and rurality level modify access to EHS beyond individual-level factors. As policy-makers in LMICs continue to make efforts to achieve the 2030 SDG goals for UHC, attention should be given to interventions that will affect contextual factors. Boosting girl child education and poverty reductions strategies were some of the suggested interventions. While implementing interventions to advance UHC, strategies targeted explicitly at uneducated mothers might help attenuate the impact of illiteracy on access to EHS in the short term. In the long-term, policies to tackle persistent cultural attitudes to girls’ education, higher rates of drop-out by girl children, and early or child marriage will be necessary to reduce suboptimal access to health services. We also suggest that future studies should include assessing interactions between contextual and individual-level variables, especially key significant ones.

###  Study Strengths and Limitations

 Our findings should be interpreted with the following caveats in mind. The data used for this study is based on a cross-sectional study design; this limits our ability to draw causal interference; therefore, caution must be observed when interpreting the data. The association between neighbourhood characteristics and inadequate access to EHS may be attributable to non-random selection of individuals into neighbourhoods rather than neighbourhood factors. As a result, these relationships should only be viewed as connections. We lacked longitudinal neighbourhood measures, and we also did not assess the length of time participants spent in their neighbourhoods or the amount to which they were exposed to the neighbourhood environment. As a result, we could not ascertain whether the relationships between neighbourhood characteristics and inadequate access to EHS were attributable to cumulative effects. The analysis employed administrative boundaries, which may not effectively capture the social context necessary for insufficient access to EHS. Lastly, we computed the outcome variables using a globally acceptable framework published by the WHO/World Bank for tracking UHC progress. However, we could not use all the indicators in the framework due to the unavailability of these variables in LMICs; this challenge was also highlighted in the WHO/World Bank report. Even with these limitations, the DHS data are usually national representative; therefore, findings from analysis are generalisable. The DHS data are typically regarded as high quality with a high response rate and sampling done with sound methodology.

## Acknowledgements

 The authors thank the MEASURE DHS project for their support and for free access to the dataset used. AAA is funded by the National Institute for Health Research (NIHR) Applied Research Collaboration (ARC) West Midlands. The views expressed are those of the author(s) and not necessarily those of the NIHR or the Department of Health and Social Care.

## Ethical issues

 This study is based on a secondary dataset from the DHS; therefore, ethical approval is not required. Data used is available in public domains.

## Competing interests

 Authors declare that they have no competing interests.

## Authors’ contributions

 SSA contributed to the study design and conceptualization and wrote the first draft of the article and analyzed the data. AAA, ORO, and OAU provided technical support and critically reviewed the manuscript for its intellectual content. All authors read and amended drafts of the paper and approved the final version.

## Supplementary files

Supplementary file 1 contains Tables S1-S3.
