# Supplementary material for: Individual and Contextual Factors Associated With Maternal and Child Health Essential Health Services Indicators: A Multilevel Analysis of Universal Health Coverage in 58 Low- and Middle-Income Countries
Source: Int J Health Policy Manag. 2021 Sep 1;11(10):2062–71. doi: 10.34172/ijhpm.2021.121 (PMC9808265; doi:10.34172/ijhpm.2021.121)
Supplement: Supplementary file 1 — contains Tables S1-S3. [file ijhpm-11-2062-s001.pdf]

Article title: Individual and Contextual Factors Associated With Maternal and Child Health Essential Health Services Indicators: A Multilevel Analysis of Universal Health Coverage in 58 Low- and Middle-Income Countries

Journal name: International Journal of Health Policy and Management (IJHPM)

Authors' information: Seun S. Anjorin[1]*, Abimbola A. Ayorinde[2], Oyinlola Oyebode[1], Olalekan A. Uthman[1]

[1]Warwick-Centre for Global Health, Division of Health Sciences, Warwick Medical School, University of Warwick, Warwick, UK.

[2]Warwick-Centre for Applied Health Research and Delivery (WCAHRD), Division of Health Sciences, Warwick Medical School, University of Warwick, Warwick, UK.

(*Corresponding author: seun.anjorin@warwick.ac.uk)

Supplementary file 1

Table S1. Description of DHS data by Countries, Survey year and percentage of Suboptimal, Average and Optimal Access to UHC health services

S/N	Country	Total	Low access	Medium access	High access
1.	Afghanistan	6,074	59.27	33.82	6.91
2.	Albania	537	56.24	43.76	0.00
3.	Angola	2,168	54.61	36.02	9.36
4.	Armenia	343	6.12	83.67	10.20
5.	Bangladesh	1,604	44.89	52.56	2.56
6.	Benin	2,511	31.46	55.08	13.46
7.	Burkina Faso	2,860	25.84	63.39	10.77
8.	Burundi	2,527	18.99	63.47	17.53
9.	Cambodia	1,411	20.41	69.03	10.56
10.	Cameroon	2,102	37.01	45.05	17.94
11.	Chad	2,941	68.38	25.71	5.92
12.	Colombia	1,677	100.00	0.00	0.00
13.	Comoros	616	32.14	52.27	15.58
14.	Congo	1,508	26.06	42.57	31.37
15.	DR Congo	3,180	40.25	41.19	18.55
16.	Cote d'Ivoire	1,288	40.76	42.24	17.00
17.	Dominican Republic	724	21.13	66.57	12.29
18.	Egypt	3,294	14.06	75.74	10.20
19.	Ethiopia	1,941	61.10	34.83	4.07
20.	Gabon	897	48.83	46.71	4.46
21.	Gambia	1,595	9.34	46.83	43.82
22.	Ghana	1,032	9.98	52.23	37.79
23.	Guatemala	2,146	24.74	63.33	11.93
24.	Guinea	1,424	56.74	31.95	11.31
25.	Haiti	1,124	51.07	38.43	10.50
26.	Honduras	1,856	5.55	73.33	21.12
27.	India	50,857	25.93	55.26	18.81
28.	Indonesia	3,544	31.09	64.90	4.01
29.	Jordan	1,980	11.77	71.97	16.26
30.	Kenya	3,584	22.18	48.30	29.52
31.	Kyrgyz Republic	862	6.61	72.85	20.53
32.	Lesotho	554	20.58	69.13	10.29

33.	Liberia	1,141	29.10	41.81	29.10
34.	Malawi	2,806	9.73	52.03	38.24
35.	Maldives	587	14.31	77.68	8.01
36.	Mali	1,966	29.70	44.81	25.48
37.	Mozambique	2,011	30.83	51.82	17.35
38.	Myanmar	905	23.09	41.44	35.47
39.	Namibia	468	17.09	65.17	17.74
40.	Nepal	1,031	22.79	69.25	7.95
41.	Niger	2,239	41.40	42.12	16.48
42.	Nigeria	6,200	45.52	35.34	19.15
43.	Pakistan	2,465	38.38	46.73	14.89
44.	Peru	1,618	21.01	72.87	6.12
45.	Philippines	1,892	29.18	66.44	4.39
46.	Rwanda	1,324	14.50	61.25	24.24
47.	Senegal	2,316	17.44	47.75	34.80
48.	Sierra Leone	1,961	22.54	46.35	31.11
49.	South Africa	282	21.99	69.86	8.16
50.	Tajikistan	1,280	16.95	63.98	19.06
51.	Tanzania	1,845	25.96	62.06	11.98
52.	Timor-Leste	1,458	27.09	40.53	32.37
53.	Togo	1,355	34.32	55.57	10.11
54.	Turkey	745	14.50	82.95	2.55
55.	Uganda	2,571	16.92	44.19	38.90
56.	Yemen	3,150	65.08	31.30	3.62
57.	Zambia	2,156	29.73	60.16	10.11
58.	Zimbabwe	990	21.92	61.31	16.77

Table S2. Multinominal Multilevel analysis of suboptimal and optimal access compared to average access in Low Income Countries

	(1)	(2)	(3)	(4)	(5)
FP1					
cons_1	0.69[0.46,1.03]	0.32[0.18,0.55]***	0.32[0.18,0.57]***	0.68[0.37,1.27]	0.43[0.23,0.81]**
Year		1.52[0.74,3.10]	1.52[0.72,3.19]	1.51[0.79,2.89]	1.48[0.77,2.83]
Age of marriage (< 18 as ref)		1.03[0.97,1.08]	1.08[1.02,1.14]**	1.12[1.06,1.19]***	1.03[0.97,1.09]
**Religion (Christianity as ref)**					
Muslim		1.25[1.15,1.35]***	1.21[1.12,1.31]***	1.34[1.23,1.45]***	1.18[1.08,1.28]***
Other religion		1.40[1.17,1.67]***	1.40[1.17,1.68]***	1.72[1.43,2.07]***	1.33[1.11,1.59]**
No religion		1.15[0.97,1.37]	1.23[1.04,1.45]*	1.31[1.10,1.56]**	1.14[0.95,1.35]
**Mother's age (14-25yrs as ref)**					
26-34		0.90[0.84,0.95]***			0.89[0.84,0.95]***
34-49		0.97[0.90,1.04]			0.97[0.90,1.05]
**Maternal Education** (Secondary education as ref)					
No education		1.64[1.50,1.80]***			1.53[1.39,1.68]***
Primary Education		1.40[1.29,1.53]***			1.39[1.27,1.51]***

**Maternal Wealth-Index** (High as ref)					
Low		1.51[1.41,1.62]***			1.32[1.21,1.44]***
Average		1.23[1.15,1.31]***			1.12[1.04,1.20]**
Media Access		0.87[0.84,0.90]***			0.89[0.86,0.92]***
Female Household-head		1.02[0.95,1.09]			1.02[0.95,1.10]
Maternal Not Working		1.10[1.04,1.17]**			1.07[1.00,1.14]*
Maternal has health insurance		0.76[0.65,0.90]**			0.74[0.62,0.88]***
**Community-level Factors**					
Community Poverty-level			1.40[1.32,1.48]***		1.18[1.10,1.26]***
Community Illiteracy Level			1.45[1.37,1.54]***		1.28[1.20,1.36]***
Community Unemployment Level			1.12[1.06,1.19]***		1.13[1.06,1.21]***
Diversity at Community Level			0.92[0.86,0.98]*		0.98[0.92,1.05]
Community Rurality Level			1.00[1.00,1.00]		1.00[1.00,1.00]
**Human Development Index** (Low as ref)					
Average				1.00[1.00,1.00]	1.00[1.00,1.00]
**Domestic Government Health Expenditure**					
Average				0.73[0.33,1.59]	0.75[0.34,1.63]
High				0.36[0.16,0.80]*	0.35[0.16,0.78]**
FP2					

cons_3	0.46[0.32,0.65]***	0.62[0.36,1.07]	0.55[1.00,1.00]*	0.52[0.27,1.02]	0.65[0.32,1.29]
Year		0.87[0.43,1.77]	0.92[0.45,1.90]	0.81[0.40,1.64]	0.78[0.38,1.59]
Age of marriage (< 18 as ref)		0.90[0.85,0.96]***	0.88[0.45,1.90]***	0.87[0.82,0.92]***	0.90[0.84,0.96]***
**Religion (Christianity as ref)**					
Muslim		1.04[0.95,1.14]	1.05[0.96,1.15]	1.04[0.95,1.13]	1.04[0.95,1.14]
Other religion		0.97[0.79,1.20]	0.96[0.78,1.19]	0.94[0.77,1.16]	0.98[0.79,1.21]
No religion		0.79[0.64,0.98]*	0.77[0.62,0.96]*	0.75[0.61,0.92]**	0.80[0.65,0.98]*
**Mother's age (14-25yrs as ref)**					
26-34		0.98[0.92,1.05]			0.98[0.92,1.05]
34-49		0.86[0.79,0.94]***			0.86[0.79,0.93]***
**Maternal Education** (Secondary education as ref)					
No education		0.82[0.74,0.90]***			0.84[0.76,0.93]***
Primary Education		0.92[0.84,1.00]*			0.92[0.85,1.01]
**Maternal Wealth-Index** (High as ref)					
Low		0.85[0.78,0.92]***			0.83[0.76,0.91]***
Average		0.91[0.84,0.97]**			0.90[0.83,0.97]**
Media Access		1.04[1.00,1.08]*			1.04[1.00,1.08]*
Female Household-head		1.05[0.97,1.14]			1.05[0.97,1.13]
Maternal Not Working		0.92[0.86,0.99]*			0.93[0.86,1.00]*

Maternal has health insurance		1.13[0.97,1.32]			1.14[0.98,1.33]
**Community-level Factors**					
Community Poverty-level			0.95[0.89,1.01]		1.04[0.97,1.13]
Community Illiteracy Level			0.85[0.80,0.91]***		0.92[0.86,0.99]*
Community Unemployment Level			0.98[0.92,1.04]		0.98[0.92,1.05]
Diversity at Community Level			1.03[0.97,1.11]		0.99[0.92,1.06]
Community Rurality Level			1.00[1.00,1.00]		1.00[1.00,1.00]
**Human Development Index** (Low as ref)					
Average				1.00[1.00,1.00]	1.00[1.00,1.00]
**Domestic Government Health Expenditure**					
Average				1.24[0.54,2.88]	1.23[0.52,2.89]
High				0.92[0.39,2.18]	0.92[0.38,2.22]
**RANDOM EFFECT MODEL**					
**Suboptimal vs Average Access**					
**Country-Level** Variance	1.93[1.22,3.04]**	1.69[1.17,2.43]**	1.75[1.19,2.59]**	1.50[1.13,2.00]**	1.50[1.13,1.99]**
**Community-level** Variance	1.52[1.45,1.59]***	1.37[1.32,1.44]***	1.40[1.34,1.47]***	1.60[1.52,1.68]***	1.43[1.36,1.50]***
**Optimal vs Average Access**					
**Country-Level** Variance	1.68[1.17,2.40]**	1.67[1.17,2.39]**	1.70[1.17,2.45]**	1.60[1.15,2.22]**	1.63[1.16,2.30]**
**Community-level** Variance	1.36[1.30,1.44]***	1.34[1.27,1.42]***	1.35[1.28,1.42]***	1.38[1.31,1.45]***	1.35[1.28,1.42]***

OD					
bcons_1	2.72[2.72,2.72]***	2.72[2.72,2.72]***	2.72[2.72,2.72]***	2.72[2.72,2.72]***	2.72[2.72,2.72]***
bcons_2	2.72[2.72,2.72]***	2.72[2.72,2.72]***	2.72***	2.72[2.72,2.72]***	2.72[2.72,2.72]***
N	32576	32576	32576	32576	32576

Table S3. Multinominal Multilevel analysis of suboptimal and optimal access compared to average access in Middle Income Countries

**FIXED EFFFECT MODEL**	**Model 1**	**Model 2**	**Model 3**	**Model 4**	**Model 5**
		RRR (95% CrI)	RRR (95% CrI)	RRR (95% CrI)	RRR (95% CrI)
**SUBOPTIMAL vs AVERAGE ACCESS TO UHC**					
Year		1.39[0.84,2.29]	1.50[0.94,2.39]	1.23[0.78,1.92]	1.18[0.75,1.85]
Age of marriage (< 18 as ref)		1.20[1.17,1.25]***	1.30[1.26,1.34]***	1.48[1.44,1.53]***	1.19[1.16,1.23]***
**Religion (Christianity as ref)**					
Muslim		1.09[1.03,1.16]**	1.10[1.03,1.17]**	1.35[1.27,1.43]***	1.03[0.97,1.10]
Other religion		0.70[0.66,0.74]***	0.68[0.64,0.72]***	0.82[0.77,0.87]***	0.67[0.63,0.71]***
No religion		1.11[0.99,1.25]	1.19[1.06,1.34]**	1.39[1.24,1.57]***	1.10[0.98,1.24]
**Mother's age (14-25yrs as ref)**					
26-34		0.97[0.94,1.00]			0.98[0.94,1.01]
34-49		1.13[1.07,1.19]***			1.14[1.09,1.20]***
**Maternal Education** (Secondary education as ref)					

No education		1.81[1.73,1.89]***			1.66[1.58,1.74]***
Primary Education		1.31[1.25,1.37]***			1.28[1.23,1.34]***
**Maternal Wealth-Index** (High as ref)					
Low		1.93[1.84,2.02]***			1.69[1.60,1.78]***
Average		1.43[1.37,1.50]***			1.35[1.29,1.41]***
**Media Access**		0.85[0.83,0.87]***			0.86[0.84,0.88]***
**Female Household-head**		0.96[0.92,1.01]			0.96[0.92,1.00]
**Unemployed mothers**		0.94[0.91,0.98]**			0.96[0.92,1.01]
**Maternal has health insurance**		0.77[0.72,0.81]***			0.77[0.73,0.81]***
**Community-level Factors**					
Community Poverty-level			1.64[1.58,1.70]***		1.13[1.08,1.18]***
Community Illiteracy Level			1.69[1.63,1.75]***		1.26[1.21,1.31]***
Community Unemployment Level			0.96[0.93,1.00]*		0.97[0.94,1.01]
Diversity at Community Level			0.93[0.89,0.97]***		0.95[0.91,0.98]**
Community Rurality Level			1.36[1.18,1.58]***		1.23[1.07,1.42]**
**Human Development Index** (Low as ref)					
hdi3_1				1.17[0.79,1.74]	1.43[0.95,2.13]
**Domestic Government Health Expenditure**					
Average				1.28[0.65,2.51]	1.06[0.53,2.11]
High				0.65[0.36,1.18]	0.59[0.32,1.09]

**OPTIMAL VS AVERAGE ACCESS TO UHC**					
Year		1.14[0.22,0.51]	1.09[0.62,1.93]	0.95[0.55,1.66]	1.01[0.58,1.75]
Age of marriage (< 18 as ref)		0.93[0.89,0.97]***	0.93[0.89,0.97]***	0.92[0.88,0.95]***	0.93[0.89,0.97]**
**Religion (Christianity as ref)**					
Muslim		0.78[0.73,0.85]***	0.74[0.69,0.81]***	0.71[0.66,0.77]***	0.80[0.73,0.86]***
Other religion		0.69[0.64,0.75]***	0.69[0.64,0.74]***	0.66[0.61,0.71]***	0.69[0.64,0.75]***
No religion		0.87[0.74,1.02]	0.82[0.70,0.97]*	0.83[0.70,0.97]*	0.86[0.73,1.01]
**Mother’s age (14-25yrs as ref)**					
26-34		0.86[0.83,0.90]***			0.86[0.82,0.90]***
34-49		0.81[0.75,0.86]***			0.80[0.75,0.86]***
**Maternal Education** (Secondary education as ref)					
No education		0.73[0.69,0.78]***			0.76[0.71,0.81]***
Primary Education		0.87[0.83,0.92]***			0.88[0.83,0.93]***
**Maternal Wealth-Index** (High as ref)					
Low		0.95[0.90,1.01]			0.89[0.83,0.95]***
Average		1.05[1.01,1.10]*			1.03[0.98,1.08]
**Media Access**		1.10[1.07,1.13]***			1.09[1.07,1.12]***
**Female Household-head**		1.07[1.01,1.13]*			1.07[1.02,1.14]*
**Unemployed mothers**		1.01[0.96,1.06]			1.00[0.94,1.06]
**Maternal has health insurance**		1.26[1.19,1.34]***			1.23[1.16,1.32]***

**Community-level Factors**					
Community Poverty-level			1.03[0.98,1.08]		1.18[1.11,1.25]***
Community Illiteracy Level			0.78[0.74,0.82]***		0.90[0.86,0.96]***
Community Unemployment Level			1.02[0.97,1.07]		1.01[0.96,1.06]
Diversity at Community Level			1.20[1.15,1.27]***		1.19[1.13,1.25]***
Community Rurality Level			0.69[0.56,0.86]***		0.75[0.60,0.94]*
**Human Development Index** (Low as ref)					
hdi3_3				0.55[0.34,0.90]*	0.52[0.32,0.85]**
**Domestic Government Health Expenditure**					
Average				0.95[0.41,2.18]	1.05[0.45,2.41]
High				0.84[0.40,1.75]	0.89[0.43,1.87]
**Suboptimal vs Average Access**					
**Country-Level** Variance	1.49[1.20,1.86]***	1.51[1.20,1.89]***	1.42[1.17,1.73]***	1.34[1.14,1.58]***	1.35[1.14,1.60]***
**Community-level** Variance	1.83[1.76,1.89]***	1.42[1.38,1.47]***	1.48[1.43,1.52]***	1.58[1.53,1.63]***	1.36[1.32,1.40]***
**Optimal vs Average Access**					
**Country-Level** Variance	1.71[1.27,2.30]***	1.90[1.33,2.69]***	1.70[1.27,2.28]***	1.56[1.22,2.00]***	1.56[1.22,1.99]***
**Community-level** Variance	2.15[2.05,2.26]***	2.19[2.08,2.31]***	2.61[2.47,2.76]***	3.09[2.91,3.28]***	2.82[2.66,3.00]***
N	95167	95167	95167	95167	95167
